# Microstructural Characterisation of Bi-Ag-Ti Solder Alloy and Evaluation of Wettability on Ceramic and Composite Substrates Joined via Indirect Electron Beam Heating in Vacuum

**DOI:** 10.3390/ma18153634

**Published:** 2025-08-01

**Authors:** Mikulas Sloboda, Roman Kolenak, Tomas Melus, Peter Gogola, Matej Pasak, Daniel Drimal, Jaromir Drapala

**Affiliations:** 1 Faculty of Materials Science and Technology in Trnava, Slovak University of Technology in Bratislava, Jána Bottu č. 2781/25, 917 24 Trnava, Slovakia; 2 The First Welding Company Inc., Kopčianska 14, 851 01 Bratislava, Slovakia; 3 FMT—Faculty of Materials Science and Technology, Technical University of Ostrava, 17. Listopadu 15, 708 33 Ostrava, Czech Republic

**Keywords:** active solder, higher application temperatures, ceramic material, electron beam soldering

## Abstract

This paper examines the wettability and interactions between ceramic and composite materials soldered with Bi-based solder containing 11 wt.% of silver and 3 wt.% titanium using indirect electron beam soldering technology. The Bi11Ag3Ti solder, with a melting point of 402 °C, consisted of a bismuth matrix containing silver lamellae. Titanium, acting as an active element, positively influenced the interaction between the solder and the joined materials. SiC and Ni-SiC substrates were soldered at temperatures of 750 °C, 850 °C, and 950 °C. Measurements of wettability angles indicated that the lowest value (20°) was achieved with SiC substrates at 950 °C. A temperature of 750 °C appeared to be the least suitable for both substrates and was entirely unsuitable for Ni-SiC. It was also observed that the Bi11Ag3Ti solder wetted the SiC substrates more effectively than Ni-SiC substrates. The optimal working temperature for this solder was determined to be 950 °C. The shear strength of the joints soldered with the Bi11Ag3Ti alloy was 23.5 MPa for the Al_2_O_3_/Ni-SiC joint and 9 MPa for the SiC/Ni-SiC joint.

## 1. Introduction

Amid significant progress in the electronic industry, there has also emerged a growing demand for advancements in the joining of ceramic and metallic materials [1]. Examples include integrated electronic components and modules connected to motherboards, as well as packaging technologies. The soldering alloy serves not only as an electrical conductor but also as a thermal conductor, while mechanically securing all the components in their proper positions [2]. Consequently, the soldering alloy must exhibit excellent electrical and thermal properties.

The primary criterion for the formation of bonds in these types of joints is effective wetting of the ceramic material [3]. However, conventional soldering methods using standard metallic solders do not enable the formation of acceptable joints with this combination of materials. To achieve reliable joints, it is essential to establish soldering conditions that allow the proper wetting of the materials being joined. This could be achieved, for example, through metallisation [4] or by the application of a suitable solder and/or brazing alloy [5].

It is the active element in the soldering alloy that reacts with the surface of the ceramic material, leading to the formation of a reaction layer [6]. However, advancements in the electronic industry also require the use of equipment capable of operating at higher service temperatures [7]. As a result, soldering alloys with a high-temperature activation of the active element were developed [8]. In such cases, soldering alloys based on zinc (Zn), tin (Sn), or bismuth (Bi) have been increasingly used [9,10,11].

Significant attention has been directed toward Bi-based solders due to their potential for use in industrial fields, where they can replace Pb-based solders [12,13]. Consequently, research has focused on developing new silver-soldering alloy materials [14,15,16] and examining the influence of silver on the properties of the resulting joints.

Titanium is the most commonly used active element in soldering alloys. Its impact on the quality of soldered and fabricated joints has been investigated in several works [17,18,19]. The application of titanium in soldering alloys has allowed the fabrication of joints between ceramic and metallic substrates. Through ultrasonic activation, joints with a high concentration of Ti at the joint interface were achieved in all the referenced cases.

The application of Bi-Ag-based solder was addressed in the work of Kolenak et al. [20], where the authors studied the soldering of Al_2_O_3_ ceramics with a Ni-SiC composite material. Bi-based solder containing 11 wt.% of silver and 1 wt.% of magnesium, with a melting point of 264 °C, was used. This solder consisted of a bismuth matrix containing silver crystals and an Ag(Mg,Bi) phase. It was observed that at the Bi11Ag1Mg/Al_2_O_3_ joint interface, a reaction layer formed due to magnesium segregation, resulting in a boundary layer approximately 2 µm thick. At the Bi11Ag1Mg/Ni-SiC joint interface, a layer formed as a result of the high silver concentration. The average shear strength of the Al_2_O_3_/Bi11Ag1Mg/Ni-SiC joint was 27 MPa.

This study focused on investigating the properties of a Bi-Ag-Ti solder and the process of soldering using the indirect electron beam heating of substrates in a vacuum. The soldering alloy was designed for applications at elevated temperatures. The titanium in the solder acted as an active element, enabling the wetting of the SiC and Ni-SiC substrates. Silver was added to the alloy to enhance its mechanical properties and electric conductivity. The shear strength of the soldered SiC and Ni–SiC joints was analysed, and for comparison, Al_2_O_3_/SiC joints were also tested to assess the potential applicability of such joints in industrial practice. This research examined the influence of alloying elements on the formation of mutual interactions between the soldered substrates and solder alloy, using microscopic analyses and wettability testing at temperatures of 750 °C, 850 °C, and 950 °C.

## 2. Materials and Methods

After determining the weight proportions of the individual components of the prepared alloy, the materials were weighed accordingly. The solder was produced using high-purity (4N) input materials. The manufacturing process was carried out in a vacuum furnace. All the weighed metals were placed in a ceramic crucible made of Al_2_O_3_. An induction vacuum furnace operating in an evacuated atmosphere was employed. Melting occurred in an argon shielding atmosphere with an overpressure of 200 mbar, using argon gas of 4.6 purity. The temperature during the solder’s production reached approximately 1100 °C. Titanium was gradually melted during the manufacturing process.

The experimental chemical composition of the solder is presented in Table 1. Chemical analysis was performed using inductively coupled plasma atomic emission spectroscopy (ICP-AES). The analysis was carried out with a SPECTRO VISION EOP instrument (SPECTRO Analytical Instruments, Kleve, North Rhine-Westphalia, Germany). Specimens for ICP-AES analysis were dissolved in appropriate acid or base solutions. The analysis was performed using an atomic emission spectrometer equipped with a pneumatic atomiser and a Scott-type sputtering chamber.

**Figure 1:**
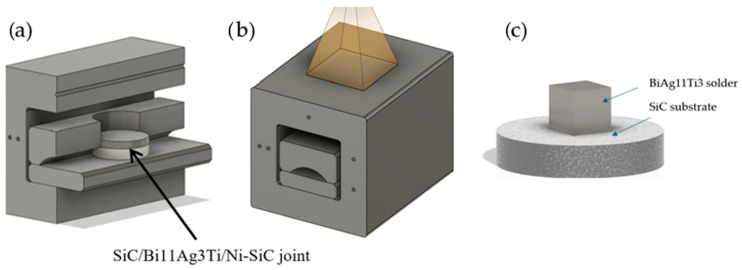
(**a**) Layout of joint specimen in graphite jig. (**b**) Scheme of heat-affected area using varying amplitudes of electron beam. (**c**) Scheme for determination of angle of wettability.

For activation of the solder and fabrication of the SiC/Ni-SiC joint—both for interaction analysis and the preparation of wettability specimens—indirect heating by electron beam was utilised. This process was carried out at FIRST WELDING COMPANY Inc., Bratislava, Slovakia, using equipment of the type PZ Elza Uni 2G. The prepared joint specimen (the preparation procedure is described in [21]) was placed into a graphite jig inside the working chamber (Figure 1a). The jig facilitated heat transfer from its surface to the joint zone. For wettability measurements, the specimens were positioned as shown in Figure 1c.

**Figure 2:**
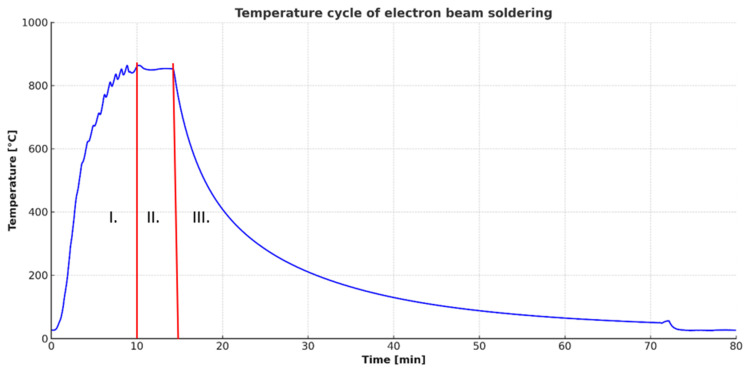
Thermal cycle of jig heating during soldering of Ni-SiC/Bi11Ag3Ti/SiC joint at temperature of 850 °C: I. heating to working temperature; II. dwell time at working temperature: III. cooling down.

The electron beam was dynamically deflected with varying amplitudes along the x and y axes to create a rectangular heat-affected area on the jig surface (Figure 1b). A type K thermocouple was attached to the jig. For the experiments focused on measuring wettability angles, a single substrate with solder applied on its surface was placed in the jig. The specimens were prepared using SiC and Ni-SiC substrates at temperatures of 750 °C, 850 °C, and 950 °C. The parameters applied during the soldering process are summarised in Table 2, along with the thermal cycle of the soldering process (Figure 2).

The following materials and dimensions were used in the experiments:

- Ceramic Al_2_O_3_ and SiC substrates in the form of discs, Ø 15 × 3 mm;
- Ni-SiC substrates in a rectangular shape, 10 × 10 mm, with a thickness of 3 mm;
- Round SiC and Ni-SiC substrates, Ø 15 × 3 mm, were used for the fabrication of joints intended for microscopic analysis.

The preparation of joint specimens and specimens for substrate wettability measurements was performed using standard metallographic procedures. The cut specimens were ground using emery paper with grit sizes of 240, 320, and 1200 grains/cm^2^. Polishing was performed on felt discs using diamond emulsions with grain sizes of 3 μm, 1 μm, and 0.25 μm.

To evaluate the suitability of the soldering alloy in terms of mechanical strength, joints were fabricated using combinations of SiC/Ni-SiC and Al_2_O_3_/Ni-SiC substrates. Two specimens from each joint type were prepared for the testing of shear strength, which was conducted using a LabTest 5.250SP1-VM machine (LABORTECH s.r.o., Opava, Czech Republic) located at the Faculty of Materials Science and Technology, Slovak University of Technology (MTF STU), Trnava, Slovakia at a loading rate of 2 mm/min until complete rupture.

Scanning electron microscopy (SEM) was employed to analyse the microstructure of the solder and the soldered joints. The investigations were performed using TESCAN VEGA 3 (Tescan Group Ltd., Brno, Czech Republic) and JEOL 7600 F (JEOL Ltd., Tokyo, Japan) microscopes, both fitted with a Microspec WDX-3PC X-ray microanalyser (Microspec Corp., Fremont, CA, USA) for qualitative and semi-quantitative chemical analysis.

The differential thermal analysis (DTA) of the Bi11Ag3Ti solder was carried out using a DTA SETARAM Setsys 18TM (KEP Technologies Inc., Austin, TX, USA). The measuring setup consisted of a cylindrical furnace with a graphite heating element, a control thermocouple, a measuring bar, and a cooling medium. Both the test and reference samples were placed in Al_2_O_3_ crucibles mounted on the measuring bar, in contact with two thermocouples, while the thermal difference between the test and reference specimens was continuously monitored.

Prior to each analysis, the inner space was purged with high-purity argon (6N) for 15 min, followed by vacuum evacuation and refilling it with argon. Throughout the analysis, a constant dynamic atmosphere was maintained in the furnace, with an argon flow rate of 2 L/h. The specimens were heated at a rate of 4 °C/min, and temperatures were recorded from room temperature up to complete melting. These temperatures were documented and analysed using SETSOFT 2000 software.

From the resulting DTA curves, the phase transformation temperatures were determined within both the liquidus–solidus range and the solid state. The enthalpies of the phase transformations were also calculated from the areas under the significant peaks.

## 3. Results

### 3.1. DTA Analysis

**Figure 3:**
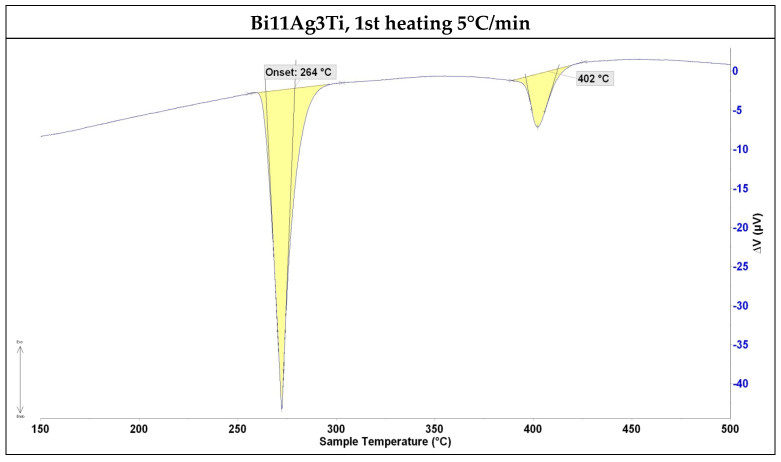
DTA analysis of Bi11Ag3Ti solder, first heating at 5 °C/min.

**Figure 4:**
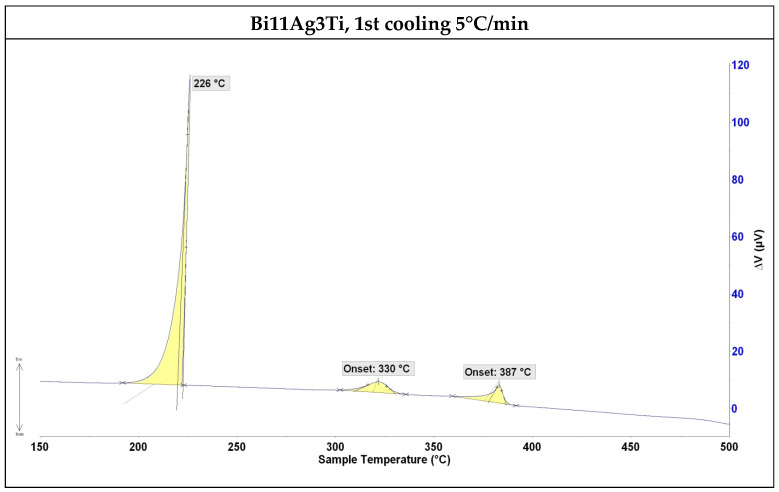
DTA analysis of Bi11Ag3Ti solder, first cooling down at 5 °C/min.

The DTA analysis (Figure 3 and Figure 4) of the solder alloy revealed the formation of silver lamellae within the bismuth matrix at a eutectic reaction temperature of 264 °C. The liquidus temperature of the alloy, recorded at 402 °C, corresponded to the binary Ag-Bi diagram. The determined phase transformation temperatures are shown in Table 3.

The primary crystallisation peak at 226 °C corresponds to the solidification of the bismuth-rich phase. The subsequent thermal events, with onset temperatures at 330 °C and 387 °C, are likely related to the precipitation of intermetallic phases or secondary solidification reactions, possibly induced by the segregation of titanium and silver within the matrix.

Titanium shows a tendency to segregate and to form intermetallic compounds or oxides (such as Bi_3_Ti_2_ or Bi_2_Ti_2_O_7_ observed in EDX point analysis), depending on the local chemical environment. Consequently, the multiple thermal events recorded during cooling are attributed not only to equilibrium phase transformations but also to the formation of metastable phases or to the precipitation of segregated elements from supersaturated solid solutions.

### 3.2. Microstructure of Soldering Alloy

**Figure 5:**
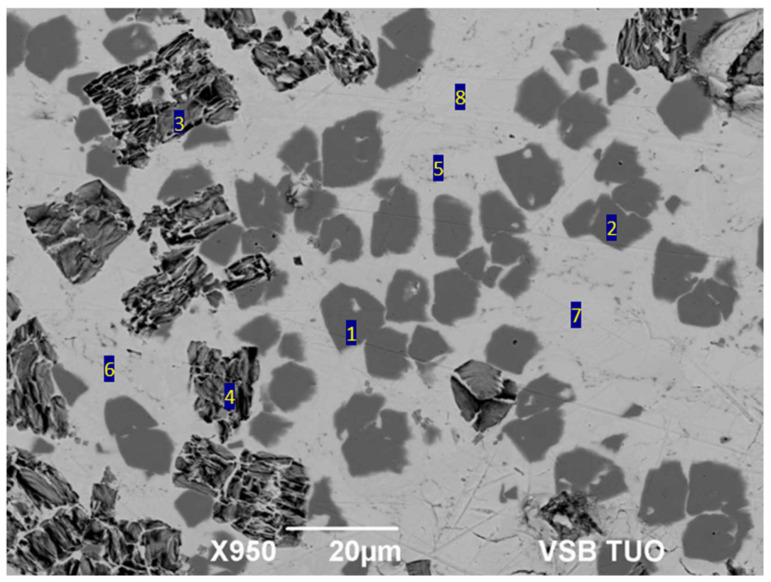
Microstructure of soldering alloy type Bi11Ag3Ti.

Point SEM/EDX analysis (Table 4) was used to determine the chemical composition of the individual components that formed the soldering alloy (Figure 5). The solder matrix (zones 5–8) consisted of a two-phase region comprising (Bi) + (Ag), while the bright zones were identified as pure bismuth. In zones 3 and 4, a Bi-rich phase containing Ti was detected. The atomic ratios suggest a stoichiometry close to Bi_3_Ti_2_, as also discussed by Weber and Rettenmayr [22]. Due to the lack of complementary phase identification techniques (e.g., XRD or XPS), the presence of oxide phases such as Bi_2_Ti_2_O_7_ cannot be confirmed, and the identification of this phase is therefore tentative. The revised interpretation considers this region as a possible Bi–Ti intermetallic compound with a high Bi content. The irregular grey constituents (Spectra 1 and 2) contained a high concentration of silver (95 at.% Ag). According to the Ag-Bi-phase binary diagram, the solidus line on the silver-rich side exhibited a retrograde character, with a maximum solubility of 3 at.% Bi at 600 °C.

### 3.3. Analysis of Transition Zone in Ni-SiC/Bi11Ag3Ti Joint

**Figure 6:**
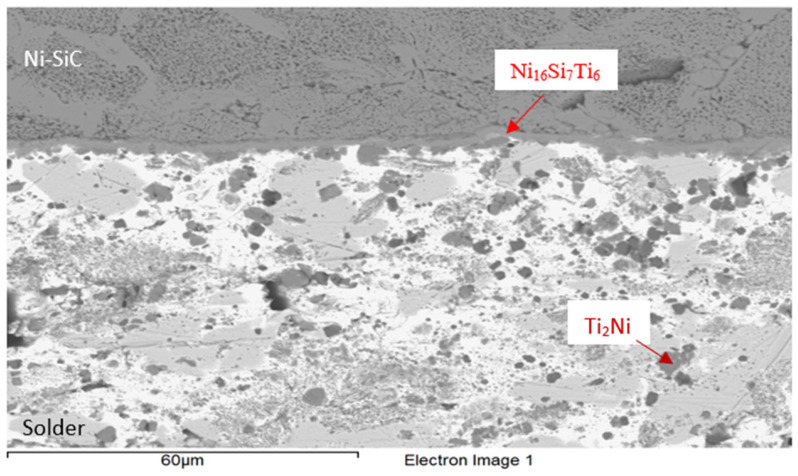
Microstructure in the boundary of Ni-SiC/Bi11Ag3Ti joint.

The concentrations of elements present at the boundary of the Ni-SiC/Bi11Ag3Ti joint (Figure 6) were determined using point EDX analysis, followed by phase identification in the boundary region. The reaction layer of the joint was formed by an intermetallic phase of the type Ni_16_Si_7_Ti_6_. Additionally, the Ti_2_Ni phase was identified within the solder. The grey zones were found to be solid solutions of silver with partially dissolved Ni, Si, and Bi elements.

**Figure 7:**
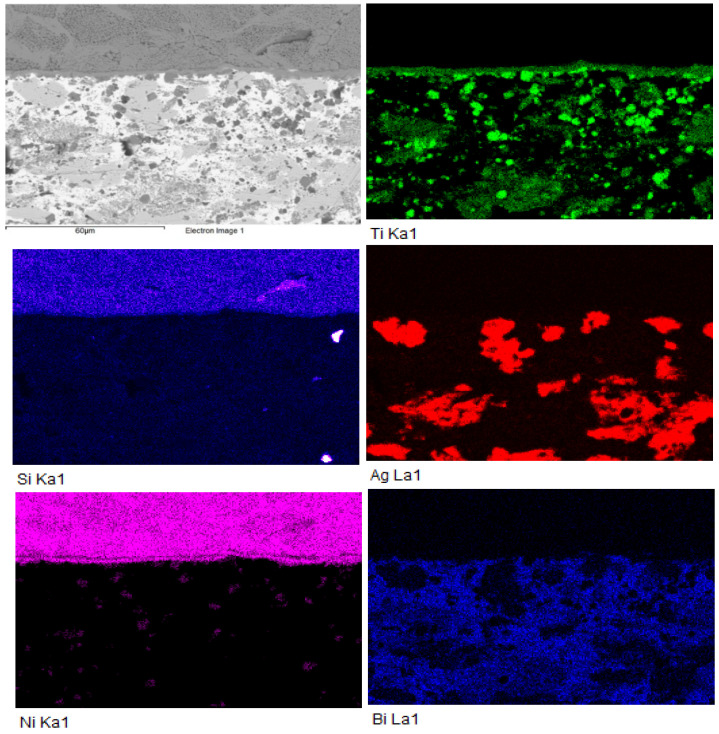
Colour map of Si, Ti, Ni, Ag, and Bi elements in boundary of Ni-SiC/Bi11Ag3Ti joint.

The colour map of elemental concentrations (Figure 7) confirmed the presence of titanium in the form of phases formed within the solder, as well as its distribution across the reaction layer zone. The diffusion of silicon into the soldering alloy and the formation of nickel islands were also observed. The silver compounds, highlighted in red, contained partially dissolved bismuth.

**Figure 8:**
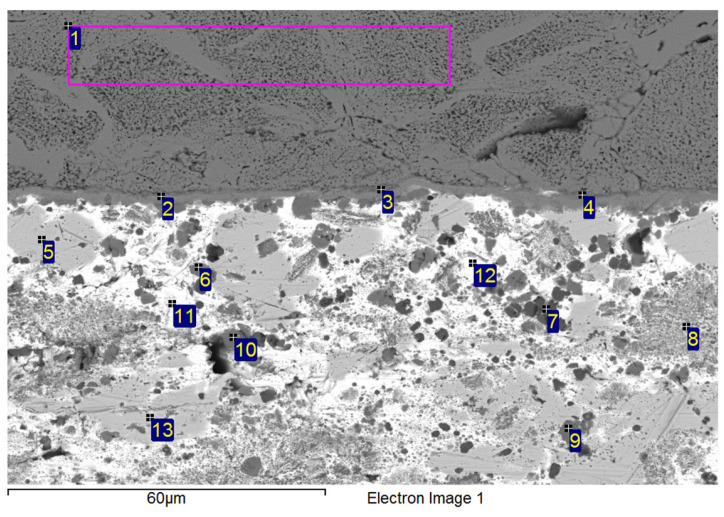
Point analysis of Ni-SiC/Bi11Ag3Ti joint boundary.

The concentrations of elements and the formed phases were studied using point EDX analysis (Figure 8) the points of measurement are designated with numbers 1 to 13. The reaction layer was composed of the intermetallic phase Ni_16_Si_7_Ti_6_. The results of the point analysis are presented in Table 5. Points 6, 9, and 10 confirmed the formation of the Ti_2_Ni phase. Zone 5 represented a solid solution of silver with partially dissolved Ni, Si, and Bi elements.

**Figure 9:**
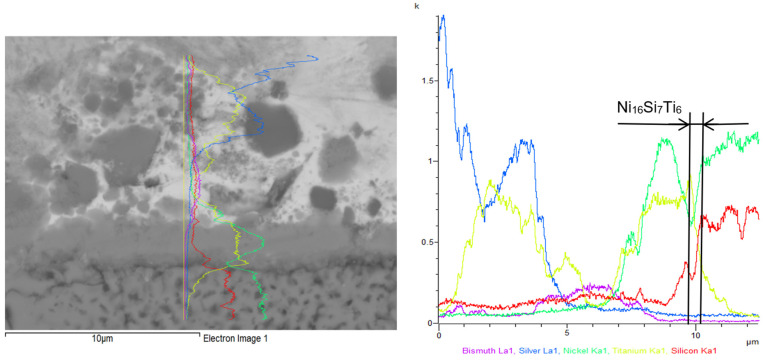
Line analysis of Ni-SiC/Bi11Ag3Ti joint boundary. The curves represent the distribution of individual elements: pink—bismuth, blue—silver, green—nickel, yellow—titanium, red—silicon.

The line analysis of the boundary between the solder and the Ni-SiC substrate (Figure 9) revealed an increased concentration of Ti, indicating the formation of the Ni_16_Si_7_Ti_6_ phase within the transition zone. A high concentration of Bi was also observed in this region, gradually decreasing towards the parent metal.

### 3.4. Analysis of Transition Zone in SiC/Bi11Ag3Ti Joint

**Figure 10:**
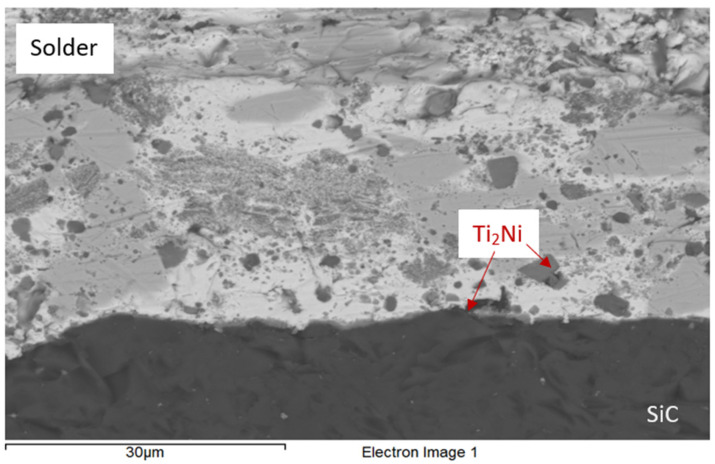
Microstructure of SiC/ Bi11Ag3Ti joint boundary.

**Figure 11:**
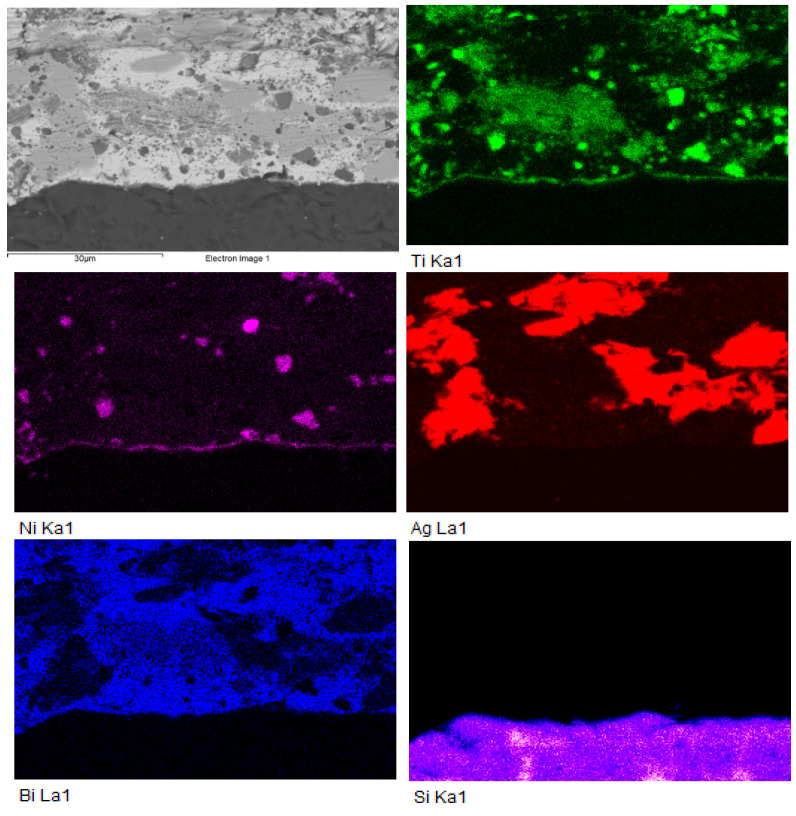
Colour map of Si, Ti, Ni, Ag, and Bi elements in boundary of SiC/Bi11Ag3Ti joint.

Figure 10 shows occurrence of Ti_2_Ni phase in solder. A planar analysis in the form of colour maps (Figure 11) indicated the distribution of Ni from the upper substrate, along with Ti concentration at the joint interface. The analysis of the SiC/Bi11Ag3Ti joint boundary confirmed the formation of a reaction layer between the SiC substrate and the solder. The intermetallic Ti_2_Ni phase was observed on the reaction layer within the joint boundary. The presence of Ni from the upper substrate demonstrated a positive interaction between the parent materials and the solder. Solid solutions of silver with partially dissolved bismuth were also present in the boundary region, marked in grey. The bright-grey areas represented solid solutions of Bi with partially dissolved silver.

**Figure 12:**
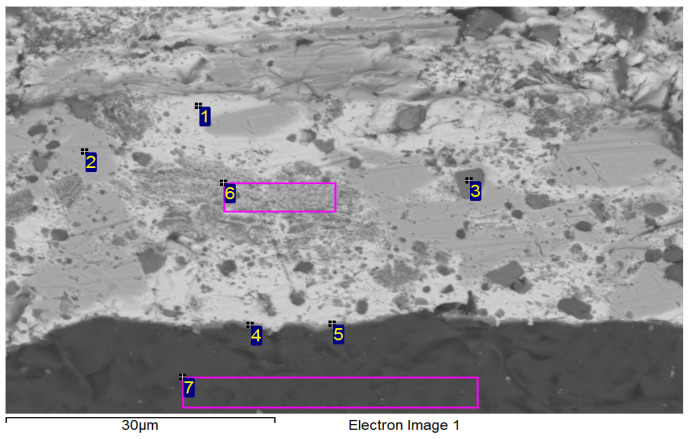
Point analysis of SiC/Bi11Ag3Ti joint boundary.

Based on the point EDX analysis (Figure 12) of seven locations designated with numbers 1 to 7 shown in the image of the studied boundary, it was found that the joint boundary was composed of the Ti_2_Ni phase. The presence of this phase confirmed the interaction between Ni from the upper substrate and the titanium contained in the solder. Table 6 provides a summary of the point analysis results. This observation was supported by the chemical composition at points 4 and 5. A solid solution of bismuth was identified at point 1, while a solid solution of silver was observed at point 2. Zone 6 depicted a mixture of Ti_8_Bi_9_ and Ti_2_Bi phases. The thickness of the reaction layer at the SiC/solder boundary was approximately 3 μm.

**Figure 13:**
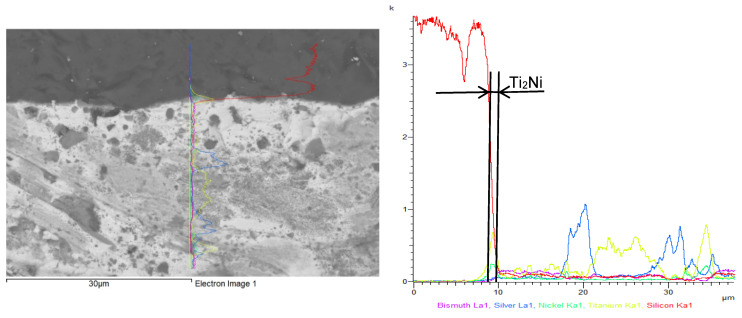
Line analysis of SiC/Bi11Ag3Ti joint boundary. The curves represent the distribution of individual elements: pink—bismuth, blue—silver, green—nickel, yellow—titanium, red—silicon.

The line analysis (Figure 13) at the boundary between the SiC substrate and the solder revealed an increased concentration of Ti in the reaction layer. Alongside Ni, the presence of the Ti_2_Ni phase confirmed that titanium had fulfilled its role as an active element, thereby promoting the formation of the soldered joint.

### 3.5. Wettability of SiC and Ni-SiC Substrates

**Figure 14:**
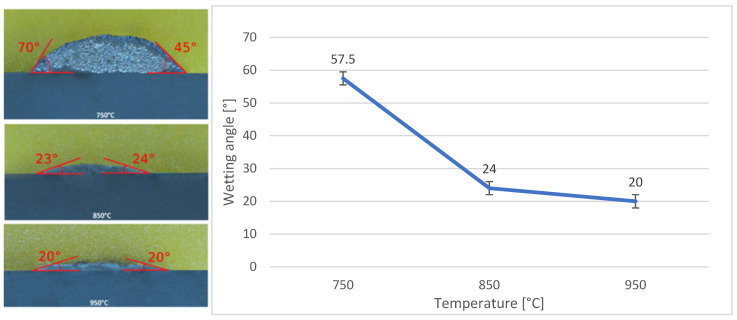
Assessment of wettability angles with Bi11Ag3Ti solder on SiC substrate.

**Figure 15:**
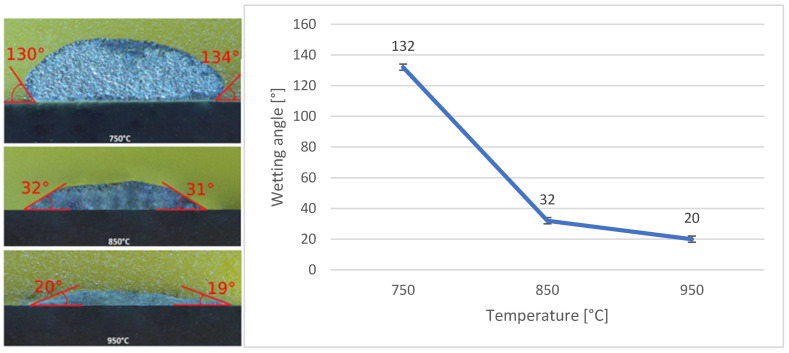
Assessment of wettability angles with Bi11Ag3Ti solder on Ni-SiC substrate.

The wettability of the substrates at three working temperatures was determined using a goniometric method (Figure 14 and Figure 15) for each soldering temperature and substrate type; two contact angles were measured and averaged to obtain a representative value. This assessment indicated that, for the SiC substrate, the wettability angle significantly improved with increasing temperatures. The most favourable wetting angle, measuring 20°, was achieved at 950 °C. Conversely, the poorest average wettability was observed at 750 °C, with a contact angle of 58°.

In the assessment of solder specimens on the composite Ni-SiC material, it was again observed that wettability had improved with increasing temperatures. The most favourable wetting angle, averaging 20°, was achieved at 950 °C. A temperature of 850 °C was also considered acceptable for the intended application, as the substrate exhibited a wettability angle of 32°, which was regarded as indicative of good wettability from a quantitative perspective. However, at a working temperature of 750 °C, the average wetting angle reached 132°, suggesting that this temperature was unsuitable for the intended application.

### 3.6. Shear Strength of Soldered Joints

**Figure 16:**
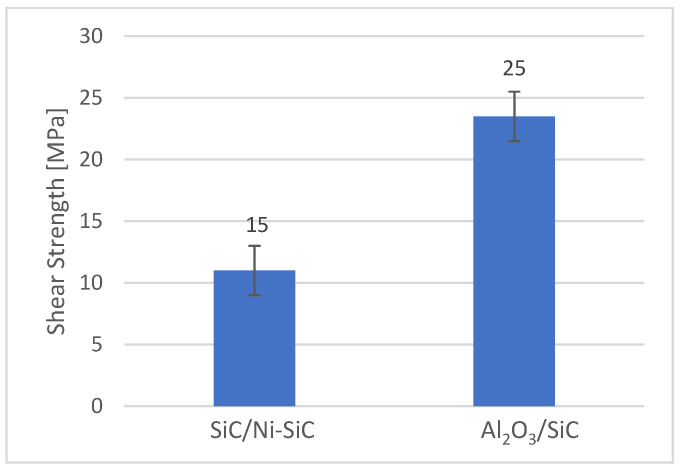
Results of mechanical shear strength test of joints fabricated with Bi11Ag3Ti alloy.

To determine the mechanical properties of soldered joints fabricated between the ceramic and composite materials using the Bi11Ag3Ti alloy, mechanical shear tests were conducted. To establish the average shear strength, three test specimens from each combination—Al_2_O_3_/Ni-SiC and SiC/Ni-SiC joints—were loaded until complete rupture. The results of the mechanical shear strength test are presented in Figure 16.

The experimental results obtained in this work were evaluated in the context of published data on Bi–Ag–Me solder systems, where Me represents active elements such as Ti, Mg, or Sb. The Bi11Ag3Ti alloy exhibited a melting temperature range of 405 °C, a shear strength between 15 and 25 MPa when joining ceramic and composite substrates, and a contact angle ranging from 20° to 132°, depending on the soldering temperature.

Comparable properties were reported for Bi11Ag1.5Ti solder, which achieved a shear strength of ~42 MPa when used on SiC/Cu joints, with similar melting behaviour [9]. Bi–Ag–Mg solders typically exhibited a lower shear strength (~27 MPa), which increased up to ~54 MPa when titanium was added, confirming the beneficial role of Ti in joint performance [20].

These results underline the competitive performance of Bi11Ag3Ti among Bi–Ag–Me solder alloys. The presence of titanium proved critical for improving both wetting behaviour and mechanical performance, particularly when joining ceramic and composite materials, due to its ability to form interfacial reaction layers.

## 4. Conclusions

The aim of this research was to determine the properties of Bi-Ag-Ti solder and to evaluate its suitability for soldering the ceramic material SiC and composite material Ni-SiC. The following results were obtained:

- The solder alloy specimen was analysed using DTA at a heating rate of 5 °C/min up to 500 °C. A peak temperature of 264 °C was observed, indicating a eutectic transformation and the formation of silver lamellae within the bismuth matrix. A second peak at 402 °C corresponded to the melting point of the soldering alloy, consistent with the binary Ag-Bi phase diagram.
- SEM/EDX analysis of the Ni-SiC/Bi11Ag3Ti/SiC joint boundary confirmed the diffusion of Ni and Si throughout the joint. These elements formed the intermetallic phase Ni_16_Si_7_Ti_6_, resulting in the formation of a reaction layer at the Ni-SiC/Bi11Ag3Ti interface. Ni was also detected at the boundary with the ceramic substrate. Ti at the joint boundary facilitated the wetting of the parent materials, leading to the formation of intermetallic Ti_2_Ni phases within the solder, along with a solid solution of Ag containing partially dissolved Bi. The reaction layer between the Ni-SiC and the solder measured approximately 5 μm in thickness.
- At the SiC/solder interface, a reaction layer approximately 3 μm thick was formed, containing the Ti_2_Ni phase. This layer indicated interaction between Ni from the upper substrate and Ti in the solder. Ti thus fulfilled its role as an active element, promoting the wetting of the parent metals and forming the observed phases at the joint boundary. A solid solution of silver was also present, with partially dissolved bismuth and the formation of a mixture of Ti8Bi9 and Ti2Bi phases.
- The wettability of the Bi11Ag3Ti solder on Ni-SiC and SiC substrates was studied at working temperatures of 750 °C, 850 °C, and 950 °C. The results indicated that both substrates achieved a wetting angle of 20° at 950 °C, which is considered indicative of good wettability. This finding also suggested that 950 °C was the most suitable temperature for use with both substrates. The measurements further showed that varying the working temperatures led to different wetting angles. This conclusion was based on the comparison of average values obtained from the tested specimens, with a clear trend of improved wettability at higher temperatures. For the Ni-SiC substrate, a working temperature of 750 °C was unacceptable.
- The mechanical shear strength test of the joint revealed a value of 15 MPa for the SiC/Bi11Ag3Ti/Ni-SiC configuration. For comparison, an Al_2_O_3_/ Bi11Ag3Ti/Ni-SiC joint was also fabricated, which achieved a shear strength of 25 MPa.
- The Bi11Ag3Ti solder alloy demonstrated a competitive performance compared to other Bi–Ag–Me systems reported in the literature. Its melting temperature range (262–405 °C), shear strength (15–25 MPa), and contact angles (20–130°) are comparable to values obtained for Bi–Ag–Ti and Bi–Ag–Mg solders. The presence of titanium significantly contributes to wetting behaviour and joint strength by promoting the formation of interfacial reaction layers on ceramic substrates. Based on these comparisons, the Bi11Ag3Ti solder demonstrates competitive performance among Bi–Ag–Me solder systems, offering a balanced combination of a low melting temperature, sufficient mechanical strength, and effective wetting on ceramic and composite substrates. These characteristics underline its potential for applications in joining dissimilar materials, especially when active element-assisted wetting is required.

## Figures and Tables

**Table 1:** Chemical composition of the Bi11Ag3Ti alloy and the results of the chemical analysis carried out by the ICP-AES method [wt.%].

Specimen	Charge [wt.%]			ICP-AES [wt.%]		
Bi11Ag3Ti	Bi	Ag	Ti	Bi	Ag	Ti
	86.0	11.0	3.0	80.4	16.9 ± 0.15	2.68 ± 0.25

**Table 2:** Working parameters of soldering process.

Accelerating voltage	55.0 kV
Current	10.0 mA
Focusing current	890.0 mA
Vacuum	1 × 10^−2^ Pa
Heating time	10 min
Heating temperature	750 °C, 850 °C, 950 °C
Time of cooling down	65 min
Distance of jig surface from the electron gun	200 ± 1 mm

**Table 3:** Temperatures of phase transformation in Bi11Ag3Ti solder, according to DTA analysis.

Composition	Onset Point on Heating (°C)		Onset Point on Cooling Down (°C)	
	First measurement	Second measurement	First measurement	Second measurement
Bi11Ag3Ti	264	263	226	330

**Table 4:** Point EDX analysis of Bi11Ag3Ti [at.%] solder.

Spectrum	O [at.%]	Mg [at.%]	Ti [at.%]	Ag [at.%]	Bi [at.%]	Compound
Spectrum 1	0	0.7	0	95.8	3.6	Ag rich
Spectrum 2	0	0.7	0	96.1	3.2	Ag rich
Spectrum 3	17.8	0	15.3	0	66.9	Bi_2_Ti_2_O_7_
Spectrum 4	17.1	0	17.6	0	65.3	Bi_2_Ti_2_O_7_
Spectrum 5	0	0	0	5.6	94.4	(Bi) + (Ag)
Spectrum 6	0	0	0	4.2	95.8	(Bi) + (Ag)
Spectrum 7	0	0	0	0	100.0	(Bi)
Spectrum 8	0	0	0	0	100.0	(Bi)

**Table 5:** Results of point analysis in Ni-SiC/Bi11Ag3Ti joint boundary.

Spectrum	Si [at.%]	Ti [at.%]	Ni [at.%]	Ag [at.%]	Bi [at.%]	
1	32.5	0	67.6	0	0	Substrate Ni-SiC
2	23.0	24.0	52.4	0	0.7	Ni_16_Si_7_Ti_6_ Ni_16_Si_7_Ti_6_ Ni_16_Si_7_Ti_6_
3	22.9	23.5	53.2	0	0.5	
4	26.2	16.9	55.9	0.2	0.8	
5	1.4	0	1.1	95.3	2.2	Solid solution (Ag) with Bi, Ni and Si
6	1.1	48.8	21.9	2.5	25.7	Ti_2_Ni
7	1.2	85.7	1.3	0.6	11.2	Ti + Ti_3_Bi
8	1.2	65.2	0.5	5.8	27.3	Heterogenous solution Ti_2_Bi+(Ag)
9	0.5	58.1	29.4	9	3	Ti_2_Ni Ti_2_Ni
10	0.8	60.6	31.4	0.4	6.8	
11	3.0	0.9	1.9	1.1	93.2	Solid solution (Bi) with Ag, Ti, Si a Ni
12	9.5	17.9	32.0	1.3	39.3	Solid solution (Bi) Ni_3_SiTi_2_
13	0.6	9.8	0	84.5	5.1	Phase Ag (85%at.) with phase Ti_2_Bi

**Table 6:** Results of point analysis in SiC/Bi11Ag3Ti joint boundary.

Spectrum	Si [at.%]	Ti [at.%]	Ni [at.%]	Ag [at.%]	Bi [at.%]	
1	0	0	0	6.7	93.3	Solid solution (Bi) with partially dissolved Ag
2	0	0	0	97.5	2.5	Solid solution (Ag) with partially dissolved Bi
3	0	67.1	32.9	0	0	Ti_2_Ni
4	25.8	40.6	21.6	0	12.0	Ti_2_Ni
5	21.5	40.9	20.0	0	17.6	Ti_2_Ni
6	0	57.5	0	0	42.5	Phases Ti_8_Bi_9_ and Ti_2_Bi
7	100	0	0	0	0	Substrate SiC

## Data Availability

The original contributions presented in this study are included in the article. Further inquiries can be directed to the corresponding author.
